# Fear of Cancer Recurrence Among Parents of Children with Cancer Who Underwent Germline Genetic Testing

**DOI:** 10.3390/curroncol33030133

**Published:** 2026-02-25

**Authors:** Emily A. Flesher, Gabrielle M. Armstrong, Jessica S. Flynn, Leila Sachner, Alise Blake, Anna M. Jones, Rachel Webster, Carolyn E. Humphrey, Niki Jurbergs, Chia-Wei Hsu, Haitao Pan, Kim E. Nichols, Belinda N. Mandrell, Katianne M. Howard Sharp

**Affiliations:** 1 Department of Psychology & Biobehavioral Sciences, St. Jude Children’s Research Hospital, Memphis, TN 38152, USAanna.jones@stjude.org (A.M.J.); rachel.webster@stjude.org (R.W.); niki.jurbergs@stjude.org (N.J.); 2 Department of Psychology, The University of Oklahoma, Norman, OK 73072, USA; 3 Department of Psychology, University of Mississippi, Oxford, MS 38677, USA; 4 Department of Oncology, Division of Cancer Predisposition, St. Jude Children’s Research Hospital, Memphis, TN 38152, USA; alise.blake@stjude.org (A.B.); kim.nichols@stjude.org (K.E.N.); 5 Department of Biostatistics, St. Jude Children’s Research Hospital, Memphis, TN 38152, USA; chia-wei.hsu@stjude.org (C.-W.H.); haitao.pan@stjude.org (H.P.); 6 Department of Pediatrics, Division of Nursing Research, St. Jude Children’s Research Hospital, Memphis, TN 38152, USA; belinda.mandrell@stjude.org

**Keywords:** fear of cancer recurrence, pediatric oncology, parent, psychosocial, spirituality, cancer predisposition

## Abstract

Fear of cancer recurrence (FCR) is a significant but understudied concern among parents of childhood cancer survivors. This study quantitatively characterized parental FCR and explored potential demographic and clinical correlates among parents of children treated for cancer. Parents (*N* = 192) completed the Fear of Cancer Recurrence Inventory-Parent Short Form (FCRI-Parent) and provided demographic information. Clinical variables were obtained from medical chart review. Associations between FCR and demographic or clinical variables were analyzed using *t*-tests, ANOVAs, and Pearson’s correlations. Parents reported a mean FCR score of 18.64 (*SD* = 8.73), with 42.2% of parents endorsing FCR above a score of 22. Parental FCR significantly varied by parent race, education, and spirituality. Higher FCR was also significantly negatively correlated with child age, time since diagnosis, and time since treatment completion. Parents of children with central nervous system tumors or hematological malignancies endorsed significantly higher FCR compared to parents of children with solid tumors. Findings build on previously identified psychosocial needs for parents of children treated for cancer by quantitatively describing parental FCR and exploring subgroups that may be at increased risk for FCR. Tailored interventions, including strategies that support spiritual coping, may help mitigate FCR among at-risk parents.

## Simple Summary

Fear of cancer recurrence is a common mental health concern among caregivers of adults with cancer, yet its prevalence and risk factors among parents of children treated for cancer are unclear. In this study, parents of children treated for cancer completed a questionnaire assessing fear of their child’s cancer recurrence. Results showed parental fear of recurrence levels similar to caregivers of adults with cancer. Fear of recurrence was associated with child age, cancer type, and time since diagnosis and treatment completion, as well as parent race, education, and spirituality. These findings suggest subgroups of parents who may be at higher risk for experiencing fear of recurrence. These results suggest that fear of recurrence may be a psychosocial need for parents of children with cancer following cancer-directed treatment. Future research should develop and validate fear of recurrence screening measures for parents, with mental health resources tailored to address these concerns.

## 1. Introduction

Fear of cancer recurrence (FCR) is one of the most prominent and continuing unmet needs for caregivers of cancer patients [1,2,3,4], referring to the worry or anxiety that cancer may return or progress [5]. Recent studies indicate that nearly half of caregivers experience clinically significant FCR [4], with rates comparable to or exceeding those observed for cancer survivors [1,2,3,4,6,7,8,9]. Caregiver FCR is associated with adverse caregiver outcomes, including poorer quality of life (QoL), planning for the future, and immune system functioning, as well as higher anxiety, depression, burnout, fatigue, cardiovascular disease, and mortality [3,4,6,10]. Although most research has focused on FCR in caregivers of *adult* cancer patients (e.g., spouses) [3], advances in childhood cancer treatment have created a growing population of childhood cancer survivors and shifted attention toward long-term quality of life [11]. Thus, FCR is increasingly relevant to the emotional wellbeing of parents of childhood cancer survivors.

Qualitative research exploring FCR among caregivers of primarily adult cancer patients highlights themes unique to the caregiver: fear of their loved one suffering or dying, a perceived need to protect the patient, and felt responsibility for the patient’s health [3,6,12]. Caregivers often experience persistent and pervasive uncertainty during and beyond the patient’s treatment, including fear of potential suffering from cancer treatment, feeling helpless while witnessing treatment-related symptoms and distress, and concern for physical symptoms with unclear etiology (e.g., recurrence, late effects), all of which can be distressing for caregivers [3,6]. Such fear and uncertainty may contribute to hypervigilance surrounding the patient’s health, protective monitoring, and heightened sensitivity to cancer-related content (e.g., media) [3,13]. Additionally, these experiences can negatively impact the caregiver–patient relationship, creating relational tension with their loved one and mistrust toward the medical system [3,6,14]. However, considerably less is known regarding FCR among parent caregivers of children with cancer.

The limited literature suggests important implications for parental FCR on their child’s healthcare utilization and pain interpretation [8,15]. Specifically, parents of childhood cancer survivors often interpret their child’s pain as a possible sign of cancer recurrence [16], with higher parental FCR associated with greater pain catastrophizing (i.e., thinking about pain in a way that is ruminative, magnifies its severity, or reflects perceived helplessness) [8,15]. Elevated parental FCR is also associated with more contact with their child’s medical providers and more frequent medical appointments [15]. Importantly, childhood cancer survivors qualitatively describe taking their cues on pain appraisal from their parents [16]. Thus, parental FCR may have significant consequences not only for parents but also for patients’ health behaviors and wellbeing.

Conceptual models grounded in the Family Stress Coping framework suggest that FCR is influenced by individual demographic factors (e.g., age, education, sex, and race) and illness-related factors (e.g., time since diagnosis) [7]. However, few studies have examined correlates of parental FCR, and the extant literature has yielded mixed findings. Although Tutelman and colleagues [8] found that parental FCR was associated with younger age, more intensive cancer treatment, and shorter time off treatment, other studies did not find significant associations between parental FCR and either child age or time since diagnosis [15,17]. In contrast, Peikert and colleagues [17] found significant associations with having fewer children, lower parental education, parent sex (i.e., higher FCR among mothers compared to fathers), and a central nervous system (CNS) tumor diagnosis. However, these findings have not yet been replicated in this population and the broader caregiver literature yields mixed findings for similar correlates [2]. Thus, there is a need for research to characterize parental FCR in this population. The broader adult survivor literature posits additional demographic and clinical correlates of FCR not yet examined in parent caregivers of pediatric cancer patients, including treatment type, ethnicity, spirituality, religious coping, family history of cancer, and a germline predisposition to cancer [2,14,18,19,20]. Germline genetic testing is increasingly integrated into pediatric cancer care through paired tumor–germline sequencing and targeted genetic counseling referrals and is rapidly becoming standard of care, with up to 18% of children with cancer found to have a cancer predisposition [21,22,23]. With cancer predisposition increasingly identified and disclosed to parents of children with cancer, children’s genetic test results may be an increasingly relevant correlate of FCR in parents of children who have already experienced cancer.

Overall, little is known regarding the prevalence and correlates of FCR among parents of children treated for cancer. Given concerning psychosocial and medical consequences of parental FCR, identifying parents at increased risk is critical. The purpose of this study was to (1) describe parental FCR and (2) explore potential demographic and clinical correlates of FCR among parents of children treated for cancer or benign tumor. Although a validated measure of FCR has been adapted for parents of childhood cancer survivors [15], the previously used clinical cutoffs, ranging from 13 to 22 [24,25,26], have not yet been validated in this population. For this reason, we described parental FCR using descriptive statistics and a conservative cutoff score to provisionally describe the proportion of parents endorsing higher levels of FCR. Parental FCR is an early and developing literature in pediatric oncology, and limited research with mixed findings preclude explicit hypotheses. Our selection of demographic and clinical correlates was informed by this limited pediatric oncology literature (i.e., parent sex, parent education, child age, treatment intensity, cancer type, and time since treatment completion), as well as conceptual models and empirical findings from the adult survivor literature (i.e., parent age at time of study, parent race, parent ethnicity, household income, religious service attendance, spirituality, relapse, subsequent cancer diagnosis, prior stem cell transplant, history of radiation therapy, genetic testing result identifying underlying cancer predisposition, and family cancer history).

## 2. Materials and Methods

### 2.1. Participants and Procedures

The current study is part of a larger, IRB-approved study at St. Jude Children’s Research Hospital (SJCRH) examining parental adjustment in the context of their child’s genetic testing result disclosure. A broad definition of pediatric cancer survivor was used consistent with the National Cancer Institute (https://cancercontrol.cancer.gov/ocs/definitions accessed on 1 November 2025), including patients who have completed treatment as well as patients continuing to receive cancer-directed treatment [27]. The study team identified potentially eligible caregivers (“parents”) in collaboration with the Division of Cancer Predisposition and verified eligibility. Inclusion criteria included parents of patients who (a) underwent germline genetic testing through SJCRH, (b) had germline results disclosed in the last 1.0 to 3.9 years, (c) had a diagnosis of a malignancy or benign tumor prior to germline testing, and (d) were fluent in spoken and written English. Parents were eligible if their child’s germline genetic testing was conducted to identify underlying cancer predisposition regardless of the approach to genetic testing, which may have included single-gene testing, panel testing, and/or whole genome and whole exome sequencing as part of genomics research (unselected for cancer diagnosis). Exclusion criteria among parents included: (a) <18 years of age, (b) having a child ≥8 years of age with significant cognitive impairment that would interfere with the child’s ability to complete questionnaires as part of the larger study, or (c) having a child whose medical status or condition precluded study participation.

Study team members contacted eligible families by phone or during a regularly scheduled SJCRH visit to assess interest in participating in the study. Participants were selectively enrolled by genetic test result as part of the larger study to ensure similar sample sizes across genetic test results (i.e., pathogenic or likely pathogenic [P/LP], variant of uncertain significance [VUS], and negative results), and patients were matched using frequency matching across genetic test result groups according to children’s age, cancer diagnosis, and treatment status (i.e., on vs. off cancer-directed treatment). Only one parent per family was invited to complete study questionnaires. Informed consent was acquired prior to engagement in any research activities. Participants completed questionnaires in person or remotely, either online or via pen-and-paper, and received a $20 gift card for completed questionnaires. Recruitment and data collection occurred between May 2021 and October 2023.

### 2.2. Measures

#### 2.2.1. Demographic and Clinical Variables

Parents reported their own and their child’s demographic information. Demographic variables collected included child age and sex, and parent age, sex, relationship to the child, race, ethnicity, education (i.e., highest degree attained), relationship status (i.e., partnered vs. not partnered), religion, frequency of religious service attendance (in past year), spirituality, and annual household income. Race response options were based on NIH-defined race categories, allowing participants to select all that applied and/or choose “Other” with a write-in option.

Trained study staff completed medical chart reviews to extract clinical variables, including: child’s cancer diagnosis date and type (i.e., CNS, solid, or hematologic), relapse (yes/no), subsequent tumor diagnosis (yes/no), germline genetic test result (i.e., P/LP, VUS, or negative), treatment status (i.e., on vs. off cancer-directed treatment), treatment type (i.e., transplant, radiation, chemotherapy, surgery), and family cancer history (i.e., number of 1st- or 2nd-degree relatives with cancer). Child’s age at initial cancer diagnosis, time since diagnosis, and time since treatment completion (for children who were off treatment) were calculated. Trained study staff also determined treatment intensity from extracted data [28].

#### 2.2.2. Fear of Cancer Recurrence

Parents completed the Fear of Cancer Recurrence Inventory-Parent Version (FCRI-Parent) [24], which was an adapted version of the Fear of Cancer Recurrence Inventory-Short Form (FCRI-SF). The 9-item FCRI-SF was designed to screen adult cancer patients for clinically significant FCR and represents the severity subscale of the full FCR scale (*r* = 0.84) [24]. The FCRI-Parent was adapted for use with parents of childhood cancer survivors by altering the language from “my cancer” to “my child’s cancer” and validated by Tutelman and colleagues [15]. The FCRI-Parent ranges from 0 to 36; however, clinical cutoff scores have not been validated for this population. Recommended cutoff scores for adult survivors have ranged from 13 to 22 [24,25,26]; thus, we conservatively and provisionally used the following to describe parental FCR for descriptive purposes: ≥22 suggesting clinically elevated FCR, 16–21 suggesting high FCR, and 0–15 suggesting low to moderate FCR [25,26]. This measure demonstrated good internal reliability in the original validation study (α = 0.83) [8] and excellent internal reliability in the current sample (α = 0.91).

### 2.3. Analyses

Descriptive statistics were used to describe FCR. Given limited prior literature, we conducted a series of bivariate analyses to explore possible correlates. *T*-tests or one-way analyses of variance (ANOVAs; categorical variables) and Pearson’s correlation coefficients (continuous variables) were used to examine whether FCR significantly varied in relation to demographic and clinical variables. ANOVAs were further explored using Tukey–Kramer post hoc tests to determine group differences using a family-wise error rate to adjust for conducting multiple comparisons. Missing data were handled using listwise deletion given minimal missing data (i.e., <5%).

Due to small numbers of participants identifying as Asian, American Indian/Alaska Native, or another race not specified by NIH categories, these race categories were combined for analyses, resulting in three race categories: White, Black, and another race. For education, “no diploma” and “GED equivalent” were combined given small sample sizes for parents reporting no diploma. Master’s and doctorate degrees were also combined to create one category reflecting graduate training.

A two-sided significance level of *p* < 0.05 was used for statistical tests, with effect sizes (i.e., partial eta squared) and 95% confidence intervals reported. Statistical analyses were performed using IBM SPSS Version 31.

## 3. Results

### 3.1. Participants

Of 199 parents who consented, 192 (96.5%) completed the FCRI-Parent. Though parents varied in education and household income, the sample was predominantly female, White, non-Hispanic, and partnered (Table 1). The majority of parents identified as Christian (68.8%). Children had a mean age of 10.73 years (*SD* = 5.37). Nearly half of the children were diagnosed with a CNS tumor (45%), followed by solid tumors (40.3%). Eighteen children (9.4%) were treated for a benign CNS or solid tumor. A subset of children were still receiving cancer-directed treatment (9.4%). Variables with missing data are noted in Table 1 with a subscript and had missing data for two or fewer participants (i.e., 0.5–1% missing).

### 3.2. Frequency of Parent FCR

Parents reported a mean FCR score of 18.64 (*SD* = 8.73, range = 0–36). Of note, Shapiro–Wilk suggested that FCR was not normally distributed (*p* < 0.01). Given that clinical cutoffs have not yet been established for parents, we provisionally used clinical cutoffs from the FCRI-SF for descriptive purposes not to be interpreted as clinical prevalence. Of 192 parents, 81 parents (42.2%) reported FCR exceeding a score of 22, 43 (22.4%) reported high FCR (i.e., 16–21), and the remaining 68 parents (35.4%) reported low to moderate FCR (i.e., <16).

### 3.3. Demographic Correlates

Six demographic variables were significantly associated with parental FCR (Table 2). Younger child age was significantly correlated with higher FCR. FCR also significantly differed by parent race, household income, education, spirituality, and frequency of religious service attendance. Post hoc comparisons using the Tukey–Kramer test revealed that parents identifying as White reported significantly higher FCR than parents identifying as Black (Table 2). Parents identifying as another non-Black race also reported significantly higher FCR than parents identifying as Black.

Parents reporting annual household income of $75,000 to $99,999 endorsed the highest FCR, whereas parents reporting annual household income ranging from $50,000–74,999 endorsed the lowest FCR. Using the lowest income range as the reference group, no post hoc comparisons were statistically significant. With regard to education, parents with an associate degree or other technical/vocational certificate endorsed the highest FCR (Table 2), with significantly higher FCR relative to parents with no diploma or GED equivalent. Parents with a bachelor’s degree also endorsed significantly higher FCR than those with no diploma or a GED equivalent. However, parents with a master’s or doctorate degree did not significantly differ from those with no diploma or a GED equivalent, nor did parents with a high school diploma.

Although frequency of religious service attendance was significantly associated with FCR, no post hoc comparisons significantly differed using Tukey–Kramer. The nonsignificant trend reflected lower FCR with increasing frequency of religious service attendance. Parents who reported being “very spiritual” endorsed significantly lower FCR relative to parents who reported being “slightly spiritual.” The small sample of parents who reported being “not spiritual at all” had the second lowest mean level of FCR and did not significantly differ from those who self-identified as “very spiritual.” Parent age, sex, ethnicity, and relationship status were not significantly related to FCR, nor was child sex.

### 3.4. Clinical Correlates

Three clinical variables were significantly associated with parental FCR (Table 3). Significant differences in FCR were observed across cancer type. Specifically, parents of children with a CNS tumor (Table 3) or hematological malignancy reported significantly higher FCR than parents of children with a solid tumor. FCR was negatively correlated with time since diagnosis and time since treatment completion such that parents endorsed significantly lower FCR as time elapsed since their child’s cancer diagnosis and treatment completion. Relapse, subsequent cancer diagnosis, transplant, radiation therapy, treatment intensity, genetic testing result, and family cancer history were not significantly related to FCR.

## 4. Discussion

This study contributes to the limited but developing literature examining FCR among parents of children with cancer by describing parental FCR and exploring potential demographic and clinical correlates. The study demonstrated high levels of parental FCR consistent with findings from the broader caregiver FCR literature [4], which is concerning given associations between caregiver FCR and poorer psychosocial and health outcomes [3,4,6,10]. Identifying parents at risk for experiencing higher FCR will be critical for developing and tailoring psychosocial services. Our findings suggest demographic and clinical factors for further study. Specifically, higher parental FCR was reported by parents identifying as White or another non-Black race, with higher education, and parenting a younger patient. Parents of children with a CNS tumor or hematological malignancy and those with more recent time since cancer diagnosis or treatment completion also endorsed significantly higher FCR. Conversely, greater self-reported spirituality was associated with lower FCR.

Parents who endorsed greater spirituality reported significantly lower levels of FCR relative to those who self-identified as being “slightly spiritual.” Notably, parents who did not identify as spiritual endorsed FCR comparable to parents who reported being “very spiritual.” Although greater spirituality may be associated with lower FCR among those who are spiritual, parents who do not identify as spiritual may use other unexamined coping strategies. Although spiritual and religious coping have been examined in relation to FCR among adult cancer survivors with similar findings [19,20], this study is among the first to quantitatively examine spirituality and religious service attendance as a correlate of caregiver FCR. Our results suggest that spiritual coping may also be relevant for parental FCR, perhaps helping to reduce or prevent FCR. Qualitative research in the adult survivor literature suggests that spiritual coping may aid in managing uncertainty and fears about death [29], which are core features of FCR. Future longitudinal research examining spirituality and spiritual coping in a more nuance manner will be important for replicating this finding and better understanding the potential role that spirituality could play for preventing or reducing parental FCR. Additionally, studying spiritual coping in conjunction with other coping approaches may clarify the relatively low levels of FCR for parents who did not identify as spiritual. There was a trend toward lower mean FCR with more frequent religious service attendance; however, this was not statistically significant. The broader religious coping literature links religious coping with lower psychological distress among caregivers of children with cancer [30,31]. Importantly, frequency of religious service attendance may reflect connection with one’s religious community, but not necessarily religious coping, which can occur outside of religious service attendance. Religious coping itself may be more relevant for future parental FCR research.

Child age was negatively associated with parental FCR, consistent with the prior study by Tutelman and colleagues [8] and studies from the adult cancer survivor literature [2,7]. However, these findings stand in contrast to nonsignificant correlations between child age and parental FCR in other studies [15,17]. One previously proposed possibility is that parents of younger children may feel greater responsibility for monitoring health and interpreting ambiguous symptoms, as noted by parent caregivers of adult cancer patients [6], leading to heightened vigilance for signs of disease recurrence. The broader FCR literature also posits that caregiver FCR may be higher when patients are younger because cancer is perceived as less normative and more disruptive to the patients’ future physical, social, and financial functioning, which may amplify uncertainty and concerns about future treatment burden [2]. However, this has not been explicitly studied among parents of childhood cancer survivors. Importantly, illness representations and appraisals are commonly cited mechanisms of FCR among adult survivors [32,33]. Future research should explore cognitive processes underlying parental FCR in relation to child age to better understand how child age may relate to parental FCR and clarify mixed findings. Of note, the association between child age and parental FCR should be interpreted within the context of the wide patient age range and overrepresentation of CNS tumors in our study.

Parents with an associate degree or technical/vocational certificate and parents with a bachelor’s degree endorsed significantly higher FCR than those with no diploma or a GED equivalent; however, parents with a master’s or doctorate degree did not endorse significantly higher FCR relative to those with no diploma or a GED equivalent. These exploratory findings suggest that parent education may be associated with parental FCR but do not support a linear relation between highest degree attained and FCR. Higher parental education has previously been associated with poorer parent quality of life for parents of children with cancer, with hypotheses that more educated parents may be more engaged in medical decision-making and information seeking (e.g., [34]). Conversely, it has also been suggested that parents with less education may have more limited awareness of nuanced cancer-related risks [34], which could reduce worry or uncertainty. Models of FCR posit that cognitive factors, such as illness perception and appraisals, promote or underlie development of FCR [32,33], offering a potential mechanism through which education may influence FCR. One unexamined possibility is a curvilinear relation. Importantly, education is complex and unexamined aspects or correlates of education will be important to study in future research (e.g., health literacy). Lastly, these findings should be interpreted within the context of a relatively small sample size for parents with no high school diploma or a GED equivalent. Although parent income was significant associated with parental FCR, no post hoc comparisons were statistically significant. Of note, mean FCR varied across income brackets with parents reporting household incomes between $75,000 and $99,999 endorsing the highest FCR. Importantly, unexamined socioeconomic factors (e.g., insurance, access to care) may be important to consider. Further research is needed to replicate associations between parental FCR and education and examine socioeconomic factors in a more nuanced manner while accounting for their complex interdependence.

FCR significantly differed across cancer type, with parents of children diagnosed with a CNS tumor or hematologic malignancy reporting higher FCR levels than those whose child had a solid tumor. Parents of children with a CNS tumor endorsed the highest FCR, consistent with findings from a study examining fear of progression [17]. Children receiving CNS-directed treatment are at greater risk for psychosocial, cognitive, and adaptive challenges (e.g., [35]), which may heighten parental worry about the impact of potential recurrence. In our sample, a subset of patients with CNS tumors only underwent partial resection; thus, one possibility is that this may have increased FCR due to concerns about tumor progression. Late effects and risk for secondary malignancies vary across cancer type in accordance with the modality, location, and intensity of treatment; thus, variability in FCR across cancer type may also reflect treatment differences and/or an unmeasured burden of physical late effects. Indeed, a previous study found that distress among parents of children with CNS tumors differed according to whether cranial radiation was received [36]. Future research should study nuanced treatment factors not captured in this study. With advances in treatment, including immunotherapy and reductions in treatment toxicity, future research should also examine parental FCR in relation to treatment with more nuance to identify subgroups at elevated risk for FCR. Time since diagnosis and time since treatment completion were negatively correlated with parental FCR, consistent with Tutelman’s and colleagues’ [8] finding that time since treatment completion was negatively correlated with parental FCR, suggesting that FCR may decline over time. Longitudinal studies are needed to clarify the trajectory of FCR and identify subsets of parents who remain at persistently high risk.

Treatment intensity, radiation therapy, stem cell transplant, relapse, and subsequent cancer diagnosis were not significantly associated with parental FCR. This contrasts findings from Tutelman and colleagues, where treatment intensity was positively associated with parental FCR [8]. Previous literature posited that more intensive treatment may increase intrusive thoughts and subsequently FCR when treatment is perceived as more threatening [8]. Indeed, cognitive processes are hypothesized to underlie FCR development [32,33]. Empirical investigation of parents’ cognitive processes and their relation to FCR may clarify mixed findings regarding treatment type and intensity. Of note, our dichotomous radiation therapy variable lacks the nuance needed to examine whether radiation location or dosage influences parental FCR, highlighting an important consideration for future research.

FCR did not significantly differ by genetic test result. This may reflect parents’ understanding that cancer predisposition increases risk for future neoplasms but not necessarily risk for relapse, though further research on parental interpretation of genetic test results is warranted. The FCRI-Parent measures FCR but not fear of future cancer diagnoses more broadly. In our study, some patients harbored cancer predisposition syndromes that were identified incidentally during broad cancer gene panel testing. Accordingly, some carried genetic risk unrelated to their initial cancer diagnosis, and, as such, parental worry may be focused on potential *new* cancer diagnoses rather than recurrence. Prior research suggests parents of children with cancer and underlying cancer predisposition endorse greater worry about future cancer risk compared to parents of children with non-clinically actionable genetic test results [37]. Thus, one possibility is that cancer-related worry is more relevant than FCR for parents of children with genetic cancer risk.

### 4.1. Clinical Implications

Although parental FCR is an early and developing literature, these findings fit within the broader literature finding psychosocial needs for parents of childhood cancer survivors, with recommended psychosocial screening [38]. Our study findings suggest that parents’ psychosocial screening should also assess for parental FCR, with a need to develop and validate FCR clinical screening measures for this population to identify parents who may benefit from targeted intervention. Findings also suggest that there may be subgroups of parents who may be at increased risk for FCR.

Despite relatively high rates of caregiver FCR [4], a recent systematic review identified few published intervention studies targeting FCR for caregivers [39], none of which had been tailored for parents of pediatric cancer survivors. Family Caregiver—Fear Of Recurrence Therapy (FC-FORT) is one intervention targeting caregiver FCR that has demonstrated good acceptability and partial feasibility criteria, indicating promise for caregiver-focused interventions [40]. However, further research is needed to tailor such interventions for parents of children with cancer. Our exploratory findings also suggest potential benefits in tailoring interventions to support spiritual coping. Hospital-based chaplaincy programs may offer an avenue for addressing spiritual coping as a strategy to mitigate FCR, while fostering opportunities to build and maintain faith communities could further support spiritual coping during and after treatment. However, given the nascency of the parental FCR literature, there is also a need for further research to inform the development of screening and intervention approaches to this population.

### 4.2. Limitations

The present study should be interpreted in the context of several limitations. First, the caregiver sample was predominantly non-Hispanic White mothers. Because caregiver distress and uncertainty may vary by gender, race, and ethnicity [41,42], our estimates may not fully represent experiences of underrepresented groups. The limited number of paternal caregivers also reduces the precision of parent sex comparisons. Future research should prioritize targeted recruitment of fathers and families from historically marginalized communities to enhance representativeness and evaluate potential disparities in parental FCR. Second, all participants were enrolled at a single pediatric oncology center with a distinctive care delivery and financing model that may increase access to resources and psychosocial care in ways that differ from other health systems. Consequently, generalizability beyond this setting is uncertain and replication across other institutions is needed. Of note, FCR was significantly higher among parents with an associate or bachelor’s degree, supporting the need to study the complexities of FCR in more socioeconomically diverse populations. With regard to generalizability, our findings should also be considered within the context of our sampling strategy and eligibility criteria (i.e., prior genetic testing to identify possible cancer predisposition). Findings may not generalize to parents who decline or are not approached for genetic testing. Our study design also resulted in overrepresentation of CNS tumors and underrepresentation of hematologic malignancies relative to the broader pediatric oncology population. Given findings of higher FCR for parents of children with a CNS tumor, the FCR scores reported may overestimate parental FCR in the broader population.

We used the National Cancer Institute’s broad definition of survivorship [27], including a subset of patients still receiving cancer-directed therapy. Although caregiver FCR during treatment has been documented, its experience and correlates may differ during treatment versus after treatment completion and this study may miss nuances specific to the latter. Religious heterogeneity also limited nuanced analyses, with few parents identifying with non-Christian religions, which may limit generalizability. Findings should also be interpreted within the context of bivariate analyses that do not account for potential interrelation among some study variables (e.g., income and relationship status; spirituality and religious service attendance). As such, associations reported may not represent independent effects. However, these findings highlight potentially relevant correlates that should be further studied. Lastly, the cross-sectional nature of our study precludes conclusions about temporal ordering or causality. Therefore, future research employing longitudinal methods is needed to clarify FCR trajectories and identify key clinical milestones that may inform timely, effective interventions.

## 5. Conclusions

Parents play an integral role in their child’s cancer treatment and survivorship; therefore, understanding parental FCR is essential for promoting optimal quality of life for both patients and their parents. This emerging literature describing FCR for parents of children with cancer builds on existing research documenting psychosocial needs for this population [38]. As such, there is a need to develop and validate clinical FCR screening measures and targeted psychosocial interventions, particularly during the completion of cancer-directed treatment. In addition to suggesting subsets of parents who may be at increased risk for FCR, we highlight directions for future research to elucidate correlates of parental FCR and develop targeted intervention strategies to mitigate the impact of FCR in this population.

## Figures and Tables

**Table 1:** Demographic and clinical characteristics (*N* = 192).

Demographic and Clinical Variables	*N* (%)	*M* (*SD*)	Range
Parent Age (years)		40.84 (8.75)	22.55–73.23
Parent Sex (Female)	166 (86.5)		
Parent Relationship to Child			
Mother ^a^	162 (84.4)		
Father ^a^	25 (13.0)		
Grandmother	4 (2.1)		
Grandfather	1 (0.5)		
Parent Relationship Status (Partnered) ^b^	141 (73.4)		
Parent Race ^b^			
White	140 (72.9)		
Black or African American	31 (16.1)		
Asian	6 (3.1)		
American Indian/Alaska Native	2 (1.0)		
More than one Race	5 (2.6)		
Another race	7 (3.6)		
Parent Ethnicity (Hispanic or Latino)	10 (5.2)		
Annual Household Income ^b^			
<$24,999	28 (14.6)		
$25,000–$49,999	42 (21.9)		
$50,000–$74,999	34 (17.7)		
$75,000–$99,999	24 (12.5)		
≥$100,000	62 (32.3)		
Parent Education ^b^			
No diploma/GED equivalent	19 (9.9)		
High school diploma	46 (24.1)		
Associate degree or other technical/vocational certificate	34 (17.8)		
Bachelor’s degree	57 (29.8)		
Master’s or Doctorate degree	35 (18.3)		
Parent Religion ^b^			
No Religion	39 (20.3)		
Roman Catholic	19 (9.9)		
Baptist	44 (22.9)		
Pentecostal	7 (3.6)		
Methodist	12 (6.3)		
Lutheran	3 (1.6)		
Presbyterian	5 (2.6)		
Episcopalian	1 (0.5)		
Non-denominational Christian	41 (21.4)		
Mormonism	2 (1.0)		
Judaism	1 (0.5)		
Muslim	2 (1.0)		
Hinduism	3 (1.6)		
Other unspecified religion	12 (6.3)		
Parent Frequency of Religious Service Attendance (Past Year) ^b^			
Never	63 (32.8)		
Once a month	40 (20.8)		
2–3 Times a month	27 (14.1)		
About once a week	44 (22.9)		
Several times a week	16 (8.3)		
Parent Spirituality ^b^			
Not spiritual at all	11 (5.7)		
Slightly spiritual	41 (21.4)		
Moderately spiritual	68 (35.4)		
Very spiritual	71 (37.0)		
Child Age (years)		10.72 (5.36)	1.64–20.62
Child Sex (Female)	105 (54.7)		
Child Age at Initial Cancer Diagnosis (years)		6.52 (5.19)	0.02–17.20
Cancer Type			
CNS Tumor	87 (45.3)		
Hematological Malignancy	28 (14.7)		
Solid Tumor	77 (40.3)		
Relapse (Yes)	40 (20.8)		
Subsequent Cancer Diagnosis (Yes)	15 (7.8)		
Treatment Type			
Surgery	149 (77.6)		
Radiation	103 (53.6)		
Chemotherapy	146 (76.0)		
Stem Cell Transplant	20 (10.4)		
Treatment Intensity			
Least Intensive	22 (11.4)		
Moderately Intensive	32 (16.7)		
Very Intensive	92 (47.9)		
Most Intensive	46 (24.0)		
Treatment Status (Off)	174 (90.6)		
Time since Treatment Completion (years) ^b^		2.88 (2.76)	0.14–16.20
Time since Diagnosis (years)		4.20 (3.53)	1.15–18.81
Genetic Test Result			
P/LP	64 (33.3)		
VUS	64 (33.3)		
Negative	64 (33.3)		
Number of 1st or 2nd Degree Relatives with Cancer		0.99 (1.07)	0–5

^a^ Includes adoptive mothers (n = 3) and fathers (n = 1); ^b^ Includes only the participants with data for the variable. Note. CNS = Central Nervous System; P/LP = Pathogenic/Likely Pathogenic; VUS = Variant of Uncertain Significance.

**Table 2:** Parent FCR compared across demographic characteristics.

Demographic Variables	Statistical Test	*M* (SD)	*F* (df)/*t* (df)	*p*	Partial η^2^	Tukey–Kramer	95% CI
Parent Sex	*t*-Test		0.36 (190)	0.721	<0.01		
Male		19.21 (7.97)					15.83, 22.60
Female		18.55 (8.86)				00.721	17.21, 19.89
Parent Race	ANOVA		7.98 (2, 189)	**<0.001**	0.08		
White		19.76 (8.21)				**<0.001**	18.36, 21.17
Black		13.11 (8.90)					10.13, 16.10
Another race		19.33 (9.18)				**0.026**	15.71, 22.96
Parent Ethnicity	*t*-Test		−1.17 (190)	0.244	0.01		
Non-Hispanic		18.81 (8.77)					17.54, 20.09
Hispanic		15.50 (7.68)				0.244	10.06, 20.94
Parent Education	ANOVA		2.65 (4, 186)	**0.035**	0.05		
No diploma/GED equivalent		12.79 (7.51)					8.93, 16.65
High school diploma		18.82 (8.32)				0.076	16.34, 21.30
Associate degree or technical/vocational certificate		20.03 (8.04)				**0.028**	17.14, 22.92
Bachelor’s degree		19.45 (9.26)				**0.029**	17.22, 21.68
Master’s or Doctorate degree		19.37 (8.52)				0.057	16.52, 22.21
Annual Household Income	ANOVA		4.70 (4, 185)	**0.001**	0.09		
≤$24,999		20.28 (9.33)					17.13, 23.42
$25,000–$49,999		17.07 (8.58)				0.526	14.51, 19.64
$50,000–$74,999		14.66 (9.43)				0.073	11.81, 17.51
$75,000–$99,999		23.67 (6.59)				0.599	20.27, 27.06
≥$100,000		19.11 (7.94)				0.974	17.00, 21.22
Parent Relationship Status	*t*-Test		−0.92 (189)	0.358	0.01		
Not Partnered		19.70 (9.46)					17.27, 22.12
Partnered		18.38 (8.39)				0.358	16.94, 19.82
Parent Spirituality	ANOVA		4.44 (3, 187)	**0.005**	<0.01		
Not spiritual at all		17.50 (7.93)				0.957	12.43, 22.57
Slightly spiritual		21.90 (8.17)				**0.004**	19.27, 24.53
Moderately spiritual		19.56 (7.98)				0.081	17.52, 21.60
Very spiritual		16.10 (9.27)					14.10, 18.09
Parent Frequency of Religious Service Attendance	ANOVA		2.49 (4, 185)	**0.045**	0.05		
Never		20.28 (7.92)					18.16, 22.39
Once a month		20.80 (8.23)				0.998	18.15, 23.45
2–3 Times a month		18.11 (8.54)				0.803	14.88, 21.34
About once a week		16.45 (9.39)				0.153	13.92, 18.98
Several times a week		15.55 (8.86)				0.277	11.35, 19.74
**Child Sex**	*t*-Test		0.61 (190)	0.544	<0.01		
Male		19.06 (8.81)					17.21, 20.91
Female		18.29 (8.69)				0.544	16.61, 19.98
**Correlation Variables**	**Statistical Test**	***r* (** **df)**		***p***			
Parent Age	Correlation	−0.06 (190)		0.396			
Child Age	Correlation	−0.20 (190)		**0.005**			

Note. η^2^ = eta-squared; CI = confidence interval. Bolding reflects statistically significant *p*-values.

**Table 3:** Parent FCR compared across clinical characteristics.

Clinical Variables	Statistical Test	*M* (SD)	*F* (df)/*t* (df)	*p*	Partial η^2^	Tukey–Kramer	95% CI
Cancer Type	ANOVA		16.42 (2, 189)	**<0.001**	0.15		
Solid Tumor		14.60 (8.61)					12.77, 16.42
CNS Tumor		21.73 (7.46)				**<0.001**	20.02, 23.44
Hematological Malignancy		20.17 (8.59)				**0.006**	17.15, 23.19
Relapse	*t*-Test		−0.53 (190)	0.599	<0.01		
Yes		17.99 (10.04)					15.27, 20.72
No		18.81 (8.38)				0.599	17.41, 20.21
Subsequent Cancer Diagnosis	*t*-Test		0.04 (190)	0.966	<0.01		
Yes		18.73 (9.65)					14.28, 23.19
No		18.63 (8.68)				0.966	17.34, 19.93
Transplant	*t*-Test		−0.87 (190)	0.387	<0.01		
Yes		17.04 (10.27)					13.18, 20.89
No		18.83 (8.55)				0.387	17.51, 20.14
Radiation Therapy	*t*-Test		0.17 (190)	0.863	<0.01		
Yes		18.74 (9.51)					17.04, 20.44
No		18.52 (7.79)				0.863	16.69, 20.35
Treatment Intensity	ANOVA		2.06 (3, 188)	0.123	0.03		
Least Intensive		15.22 (7.27)					11.58, 18.87
Moderately Intensive		20.53 (7.29)				0.124	17.51, 23.55
Very Intensive		19.23 (8.77)				0.211	17.45, 21.02
Most Intensive		17.78 (9.85)				0.667	15.26, 20.39
Genetic Test Result	ANOVA		0.70 (2, 189)	0.500	0.01		
Negative		17.75 (8.48)					15.59, 19.90
P/LP		18.61 (9.54)				0.844	16.45, 20.76
VUS		19.57 (8.16)				0.467	17.41, 21.73
**Correlation Variables**	**Statistical Test**	***r* (** **df)**		***p***			
Time Since Diagnosis	Correlation	−0.24 (190)		**0.001**			
Time Since Treatment Completion	Correlation	−0.22 (171)		**0.004**			
Number of 1st or 2nd Degree Relatives with Cancer	Correlation	0.12 (190)		0.110			

Note. CNS = Central Nervous System; P/LP = Pathogenic/Likely Pathogenic; VUS = Variant of Uncertain Significance; η^2^ = eta-squared; CI = confidence interval. Bolding reflects statistically significant *p*-values.

## Data Availability

The data presented in this study are available on request from the corresponding author.
